# Minimally Invasive Sacroiliac Joint Fusion for the Treatment of Brucella Pyogenic Sacroiliitis: A Case Report

**DOI:** 10.7759/cureus.6212

**Published:** 2019-11-21

**Authors:** Gustavo Anton, Doris Tong, Tania Little, Teck M Soo

**Affiliations:** 1 Neurosugery, Ascension Providence Hospital, Michigan State University, College of Human Medicine, Southfield, USA; 2 Neurosurgery, Ascension Providence Hospital, Michigan State University, College of Human Medicine, Southfield, USA; 3 Infectious Disease, Ascension Providence Hospital, Michigan State University, College of Human Medicine, Southfield, USA

**Keywords:** sacroiliac joint, sacroiliitis, pyogenic sacroiliitis, brucella, sacroiliac joint fusion

## Abstract

A 39-year-old male presented with a two-month history of right hip pain. Computed tomography (CT) scan demonstrated right sacroiliac joint space widening with cortical destruction and erosive changes in the iliopsoas muscle. Minimally invasive right sacroiliac joint fusion was performed with biopsy and aspirate, which confirmed positive Brucella cultures. The patient was started on long-term antibiotic therapy, and his pain significantly improved. Pyogenic sacroiliitis is a rare condition that requires a high index of suspicion. In this case, minimally invasive sacroiliac joint fusion successfully treated the patient’s pain and instability as well as aided in the diagnosis of Brucella infection.

## Introduction

Brucella is a rare genus of gram-negative bacteria to be found in the United States; yet, it can commonly be found in cases of sacroiliitis [1-2]. Infection of the sacroiliac (SI) joint is rare, with an incidence of 1%-2% [3]. The diagnosis is often delayed because of misleading symptoms that mimic disorders of the hip and lumbosacral spine. Antibiotic therapy is the standard of care for such infections but is often unsuccessful as a stand-alone treatment. In certain cases, surgical intervention is indicated in patients who failed conservative treatment or developed abscess, bone destruction with instability, septicemia, or neurological symptoms and deficits [4]. At the time of writing this manuscript, two cases found within the literature described the use of minimally invasive sacroiliac joint fusion for the treatment of sacroiliitis, making the approach plausible for the treatment of pyogenic sacroiliitis [5-6]. Open debridement and fusion through an anterior or posterior approach for pyogenic sacroiliitis have been described in the past, with good results [3]. In this case, we are presenting a minimally invasive sacroiliac fusion as an alternative surgical approach for the treatment of pyogenic Brucella sacroiliitis.

## Case presentation

A 39-year-old male presented to our institution with a two-month history of right hip pain and difficulty ambulating. The patient reported using crutches and a walker due to the severe pain when ambulating. The patient immigrated to the United States from Cameroon in 2009. The patient later returned to Cameroon, where he spent two years in a dairy farm taking care of livestock. He subsequently developed intermittent subjective fevers of 103°F as well as painful joints. On physical exam, he had 5/5 strength in all muscle groups of his bilateral upper extremities, 3/5 strength of the bilateral hip flexors, and 5/5 in all other muscle groups in the bilateral lower extremities. Sensation was intact in all dermatomes. The patient was febrile with a maximum temperature of 101.7°F. There was no leukocytosis. Lactic acid was normal. Blood cultures were negative, and C-reactive protein (CRP) was elevated at 157.9 mg/L.

**Figure 1:**
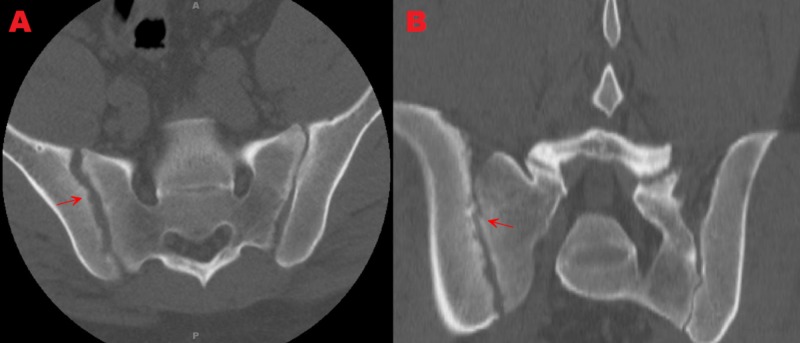
Axial and coronal computed tomography (CT) views showing right sacroiliac joint cortical destruction, erosive changes, and widening. Figure 1a: Axial view showing sacroiliac joint cortical destruction, erosive changes, and widening. Figure 1b: Coronal view showing sacroiliac joint cortical destruction, erosive changes, and widening.

**Figure 2:**
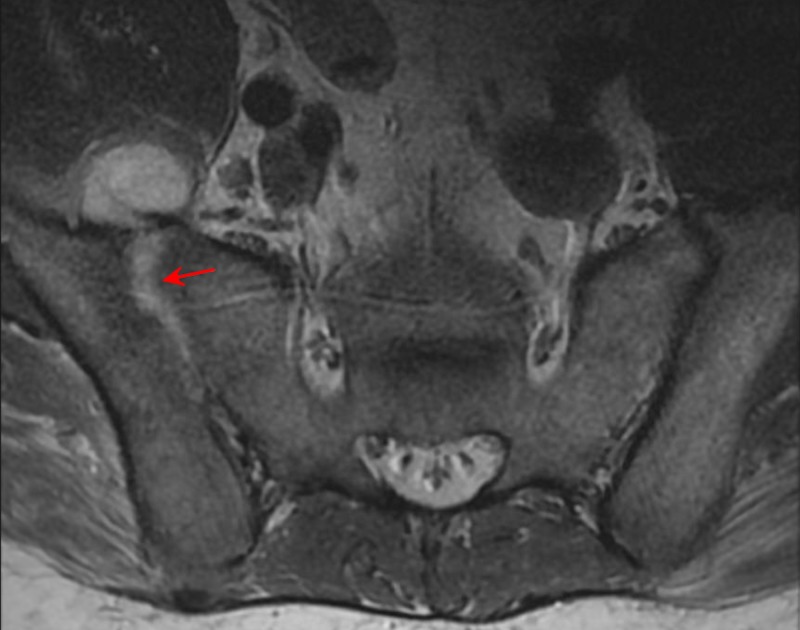
Preoperative magnetic resonance imaging (MRI) displaying right sacroiliac joint effusion with edema

Computed tomography (CT) demonstrated right sacroiliac joint space widening with cortical destruction and erosive changes involving the right sacroiliac joint when compared to the left. Fluid collection extending from the anterior aspect of the right SI joint into the iliopsoas muscle (Figure 1A, Figure 1B). Magnetic resonance imaging (MRI) revealed right sacroiliac joint effusion with extensive edema involving the iliac bone, sacrum, and iliacus muscle (Figure 2). CT-guided right sacroiliac joint aspiration was attempted, which resulted in small amounts of the specimen without frank pus. Subsequent cultures from the specimen were negative for several days. Intravenous rocephin 2000 mg q24h, vancomycin 2000 mg q12h, and oral doxycycline 100 mg q12h were started. Due to the patient’s findings, negative cultures, a small amount of specimen on CT-guided aspiration, and, most importantly, instability of the joint, minimally invasive sacroiliac joint fusion with further biopsy of the SI joint was performed.

A pin was placed on the opposite (left) posterior superior iliac spine (PSIS) to support the reference frame used in navigation. Intraoperative CT was performed to obtain data acquisition. The SI joint was identified and decorticated. Purulent material within the SI joint was aspirated and sent for microbiological and histologic studies. Under Stealth navigation guidance, the SI fusion was carried out using the Medtronic Rialto™ SI Fusion System (Medtronic, MN, USA). On the first postoperative day, the patient was able to transfer to a chair from his bed without difficulty, was able to ambulate more than 50 feet, and reported a significant reduction of pain.

Tissue and blood cultures were sent to the state department of health and human services for further testing due to the high risk of Brucella exposure due to the patient’s history. A Brucella infection was confirmed. The patient was then started on a six-week course of oral doxycycline and streptomycin after discharge and was instructed to follow up with infectious disease.

**Figure 3:**
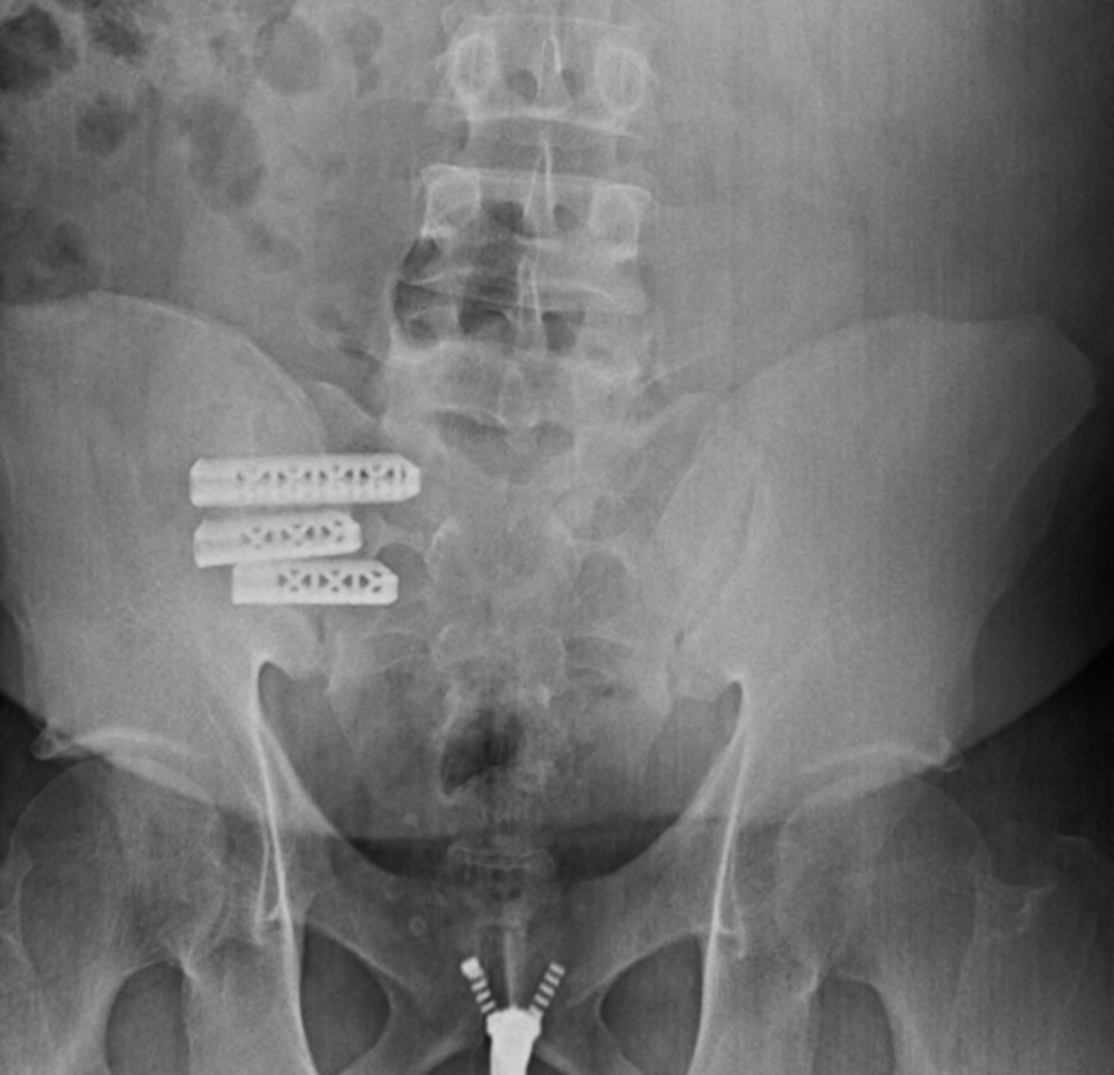
Six-month anteroposterior X-ray displaying the correct placement of implants

At the three-month follow-up, the patient reported a significant improvement in pain and ambulation after surgery. He reported intermittent pain during prolonged periods of ambulation but did not need medications to aid his pain. Since he had significant improvement, he decided to discontinue the use of pain medications. At the six-month follow-up, the patient reported complete resolution of pain. Imaging at this appointment also revealed the satisfactory placement of implants (Figure 3). Follow-up with infectious disease revealed Brucella antibodies went from a titer of 1:640 in-hospital to 1:160 at the six-month follow-up evaluation.

## Discussion

Sacroiliac joint infection is rare, and in many cases, it is usually associated with multiple predisposing factors such as intravenous drug abuse, immune suppression, pregnancy, trauma, and infections elsewhere in the body [3]. The reported incidence varies from 1%-2% of septic arthritis or osteomyelitis [3,5]. The most common organisms found in sacroiliitis are *Staphylococcus Aureus*, coagulase-negative *staphylococci*, *streptococci*, and *Pseudomonas aeruginosa,* which collectively accounted for 88% of cases [6]. In patients with Brucella infection, osteoarticular involvement is the most common presentation. Sacroiliitis in Brucella is reported to be the presenting symptom in 21%-55% of Brucellosis cases [2]. The main vector of infection is through the ingestion of unpasteurized dairy products of infected animals such as milk and cheese [5]. Other less common routes of transmission to humans include the contamination of cuts and abrasions, inhaled aerosols, and conjunctival inoculation by infected animal secretions.

Pyogenic sacroiliitis can present with buttock pain, limping, and in severe cases, patients may have gait abnormalities and inability to ambulate. The diagnosis is frequently delayed due to the presentation of non-specific and poor localizing symptoms, which mimic hip disease, osteitis of the ilium, and, in some cases, lumbar disc herniation [3]. The natural history of pyogenic sacroiliac joint infection is thought to occur most commonly by hematogenous spread, beginning with the widening of the joint space, which can be seen on radiographic studies. Aspiration of the joint should always be considered in patients with negative blood cultures and who have clinical and radiographic features suggestive of pyogenic sacroiliitis [5]. The optimal treatment of pyogenic sacroiliitis is a combination of early diagnosis, bed rest, and empirical antibiotic therapy before the causative organism is known. However, in many cases, antibiotic therapy alone may not be adequate due to poor penetration. In patients who fail conservative treatment, surgical treatment of pyogenic sacroiliitis includes debridement or debridement with arthrodesis [2]. MRI has been proven to be the best study for the early diagnosis of pyogenic sacroiliitis infection. Acute findings for infected SI joint are subchondral bone marrow edema, post-gadolinium enhancement, and soft tissue edema. Chronic findings are periarticular bone marrow reconversion, replacement of articular cartilage by pannus, bone erosion, subchondral sclerosis, and joint space widening [4].

Open SI joint fusion has been used to relieve pain from degenerative sacroiliitis or for sacroiliac joint disruption [3]. Two manuscripts within the literature reported that minimally invasive SI joint fusion was superior over the traditional open approach, with decreased intraoperative blood loss, operative time, hospital stays, and better pain relief [7-8].

## Conclusions

Sacroiliac joint infection is a frequently missed diagnosis that requires a high index of suspicion. This case report is the first reported case of Brucella sacroiliitis that was successfully treated with a combination of minimally invasive sacroiliac joint arthrodesis and antibiotics. The minimally invasive approach significantly improved the patient’s speed of recovery and demonstrated a favorable long-term outcome. We propose antibiotics in conjunction with minimally invasive sacroiliac joint fusion as an alternative to the current approaches of antibiotic therapy and/or open approach sacroiliac joint fusion in the treatment of pyogenic sacroiliitis.
